# Enhancing Detection of Medullary Thyroid Cancer in Cytologically Indeterminate Thyroid Nodules: The Role of Molecular Testing

**DOI:** 10.1016/j.aed.2026.02.004

**Published:** 2026-02-17

**Authors:** Thien Duy Nguyen, Jordan P. Reynolds, Shweta Agarwal, Marius N. Stan, Ana-Maria Chindris

**Affiliations:** 1 Department of Cancer Biology, Mayo Clinic Florida, Jacksonville, Florida; 2 Department of Pathology, Mayo Clinic Florida, Jacksonville, Florida; 3 Department of Pathology, Mayo Clinic Florida, Jacksonville, Florida; 4 Division of Endocrinology and Metabolism, Mayo Clinic Rochester, Rochester, Minnesota; 5 Division of Endocrinology and Metabolism, Mayo Clinic Florida, Jacksonville, Florida

**Keywords:** parafollicular C-cell, medullary thyroid carcinoma, atypia of unknown significance, molecular testing, serum calcitonin, fine needle aspiration

## Abstract

**Background/Objective:**

Medullary thyroid cancer (MTC) is a rare neuroendocrine tumor arising from the parafollicular C-cells. Unlike follicular-derived thyroid cancers, the only curative treatment modality for MTC is complete surgical resection which makes early detection essential. Fine-needle aspiration biopsy of the thyroid is an accurate preoperative diagnostic method; however, morphologic heterogeneity, sample quality and cytomorphologic overlap with other entities may result in misclassification, with potential for delays in treatment and poorer outcomes.

**Case Report:**

Retrospective review of 4 cases with histologically confirmed MTC, in which the initial cytological diagnosis was not indicative of MTC.

**Discussion:**

Four patients diagnosed with MTC within the past 6 years are included in this report. The age at diagnosis ranged from 36 to 64 years. Thyroid ultrasound demonstrated a solitary nodule in 3 cases and a multinodular thyroid in one. Fine needle aspiration cytology was signed off as suggestive of oncocytic (Hürthle) cell neoplasm in 2 cases, atypia of undetermined significance in one and suggestive of follicular neoplasm in the fourth case. The diagnosis of MTC was established by molecular testing in all patients, and preoperative serum calcitonin was consistent with the diagnosis. All patients had histologically confirmed MTC.

**Conclusion:**

This case series highlights the heterogenous presentation of MTC in cytology samples. In cases with indeterminate cytology, additional diagnostic tools, particularly molecular testing, should be considered to improve diagnostic accuracy and surgical management.

**Highlights**

• The rarity of medullary thyroid cancer (MTC) associated with the variable cytomorphologic appearance on fine needle aspiration cytology (FNAC) samples contribute to the challenges in its diagnosis
• According to studies, the pooled detection rate of MTCs on FNAC is about 56%, with most being misclassified as oncocytic or follicular neoplasms
• Additional ancillary testing, with molecular testing emerging as the preferred modality, should be considered to improve diagnostic accuracy and guide tailored surgical management
• Due to the recent advances in understanding the genetic alterations that drive thyroid cancers and the development of targeted therapies, the information provided by molecular testing may have additional therapeutic implications

**Clinical Relevance**

Given the known challenges in diagnosing medullary thyroid cancer in cytology samples, with potential detrimental effects on patient outcomes, the use of complementary testing should be considered in indeterminate cases, to increase diagnostic accuracy.

## Introduction

Medullary thyroid carcinoma (MTC) is a rare neuroendocrine tumor derived from calcitonin-producing parafollicular C-cells of the thyroid gland. While MTC represents less than 3% of the thyroid cancers in the United States, it accounts for a disproportionately high 13% of thyroid cancer-related mortality.^1^^,^^2^ The clinical outcome of MTC is largely associated with the stage at diagnosis. The 10-year survival decreases from 90% to 100% for tumors limited to the thyroid, to 40% for those with local metastatic disease and less than 20% of those with distant disease, underscoring the importance of timely diagnosis and treatment.^1^^,^^3^ About 25% of MTC are familial with the remainder being sporadic and typically diagnosed in the fourth to 6th decade of life.^1^^,^^2^ Sporadic MTC presents clinically as a neck mass, or as a thyroid nodule on neck imaging.^1^^,^^2^^,^^4^ The initial assessment of a thyroid nodule includes ultrasonography, followed by fine needle aspiration cytology (FNAC) if certain morphology and size criteria are met.^4^^,^^5^ The rarity of MTC combined with variable cytomorphologic appearance on FNAC samples poses significant diagnostic challenges.^6^, ^7^, ^8^ Recognizing these limitations and the inherent negative impact on patient outcome, several complementary diagnostic tools have been proposed but none widely adopted to date. In this report, we present 4 cases emphasizing on the pitfalls in the cytologic diagnosis of MTC and summarize the ancillary testing modalities -most notably molecular testing platforms-that can aid in its diagnosis.

## Case Summary

Four patients with a histologic diagnosis of MTC are presented in this case series. Three patients were women, one was male. Age at diagnosis ranged between 36 and 64 years. Initial clinical presentations included an asymptomatic thyroid nodule incidentally identified on unrelated imaging (2 cases), throat discomfort (one case) and a clinically palpable neck mass (one case). The thyroid ultrasound demonstrated a solitary nodule (3 patients) and multiple nodules (one patient). FNAC was pursued in accordance with the American Thyroid Association (ATA)/American College of Radiology criteria in 3 cases, and in one case was performed at the patient request. One of the patients, presenting for a second opinion, had an FNAC in another institution, performed 4 months earlier which was reported as atypia of undetermined significance (AUS).

The cytology results were signed off as “cytologic features suggestive for oncocytic (Hürthle) cell neoplasm” in 2 cases, AUS in one case, and “cytologic features suggestive of follicular neoplasm” in one case. In all 4 cases, the molecular testing platform ThyroSeq v3 detected gene abnormalities consistent with MTC and identified mutations in the *RET* gene in 2 cases, *HRAS* in one case, and *KRAS* in one case. Preoperative serum calcitonin was obtained in all 4 patients to determine if additional cross-sectional imaging should be performed preoperatively and ranged between 108 pg/mL and 5080 pg/mL (reference range ≤14.3 pg/mL). All patients underwent total thyroidectomy with neck exploration, and the final pathology result confirmed MTC with a tumor size range of 0.6 cm–3.0 cm. A synchronous papillary thyroid microcarcinoma was diagnosed in one case. The details of imaging, pathology, surgical, and clinical outcomes are summarized in Table 1.

**Table:** Clinical, Pathologic, and Surgical Characteristics of Patients With Medullary Thyroid Carcinoma

	Case #1	Case #2	Case #3	Case #4
Age at diagnosis (y)	58	36	64	54
Clinical presentation	Frequent throat clearing and a sensation of “something stuck in the throat”	“Lump” on the left neck noted on self-examination, without associated symptoms	Asymptomatic Thyroid nodules incidentally noted on unrelated imaging	Asymptomatic Thyroid nodules incidentally noted on unrelated imaging
Thyroid ultrasound	Thyroid gland normal in size Thyroid nodule: Right mid pole 0.8 x 0.7 × 0.8 cm Solid Hypoechoic Lobulated margins No morphologically abnormal cervical lymph nodes	Thyroid gland normal in size Thyroid nodule: Left mid-upper pole 2.3 x 1.7 × 1.4 cm Predominantly cystic Regular margins Hypoechoic solid component with small echogenic foci No morphologically abnormal cervical lymph nodes	Thyroid gland normal in size Thyroid nodule: Right mid to lower pole 1.2 x 0.7 × 1.1 cm Solid Hypoechoic Irregular margins Extrathyroidal extension Microcalcifications Peripheral macrocalcification No morphologically abnormal cervical lymph nodes	Thyroid gland with a mildly enlarged right lobe 4.7 cm × 2.7 cm x 3.2 cm Thyroid nodules: #1 Right mid pole 3.6 x 2.4 × 2.8 cm Solid Isoechoic Regular margins Peripheral macrocalcification #2 Left upper pole 0.6 x 0.6 × 0.6 cm Solid Hyper/isoechoic Regular margins Peripheral macrocalcification No morphologically abnormal cervical lymph nodes
Cytological findings	Suspicious for oncocytic (Hürthle) cell neoplasm (Bethesda category IV)	Suspicious for oncocytic (Hürthle) cell neoplasm (Bethesda category IV)	Atypia with oncocytic (Hürthle) cell changes, suggestive of lymphocytic thyroiditis (Bethesda category III)	Suspicious for follicular neoplasm (Bethesda category IV)
Molecular testing	Platform: ThyoSeq v3 Gene mutation: *HRAS* Gene fusions: neg CN alterations: neg Gene expr. profile: neg Medullary C cells: positive	Platform: ThyoSeq v3 Gene mutation: *RET* (pM918T, c2753T>C) Gene fusions: neg CN alterations: neg Gene expr. profile: neg Medullary C cells: positive	Platform: ThyoSeq v3 Gene mutation: *RET* (pC620R, c1858T>C) Gene fusions: neg CN alterations: neg Gene expr. profile: neg Medullary C cells: positive	Platform: ThyoSeq v3 Gene mutation: *KRAS* Gene fusions: neg CN alterations: positive Gene expr. profile: neg Medullary C cells: positive
Preoperative laboratory testing	Calcitonin 251 pg/mL (normal range ≤14.3 pg/mL^a^) CEA 14.8 ng/mL (normal range ≤3.0 ng/mL)	Calcitonin 108 pg/mL (normal range ≤14.3 pg/mL^a^) CEA 13.4 ng/mL (normal range ≤3.0 ng/mL)	Calcitonin 257 pg/mL (normal range ≤14.3 pg/mL^a^) CEA 23.3 ng/mL (normal range ≤3.0 ng/mL)	Calcitonin 5408 pg/mL (normal range ≤14.3 pg/mL^a^) CEA 482.2 ng/mL (normal range ≤3.0 ng/mL)
Surgical management	Total thyroidectomy	Total thyroidectomy	Total thyroidectomy and central neck dissection	Total thyroidectomy with bilateral central neck dissection
Histopathologic Diagnosis	Focality: unifocal Tumor site: right lobe Tumor size: 0.6 cm Histologic type: MTC Margins: uninvolved Angioinvasion: no Lymphatic invasion: no ETE: no Regional LN: Involved: 0 Examined: 1 AJCC staging: T1aN0Mx Immunostaining: positive for calcitonin and chromogranin Congo red stain negative for amyloid.	Focality: unifocal Tumor site: left lobe Tumor Size: 2.0 cm Histologic type: MTC Margins: uninvolved Angioinvasion: no Lymphatic invasion: no ETE: no Regional LN: Not submitted AJCC staging T1bNxMx Immunostaining: positive for calcitonin. Congo red stain focally positive for amyloid	Focality: unifocal Tumor site: right lobe Tumor Size: 1.2 cm Histologic type: MTC Margins: uninvolved Angioinvasion: no Lymphatic invasion: no ETE: no Regional LN: Involved:0 Examined: 2 AJCC staging T1bN0Mx Immunostaining: positive for calcitonin synaptophysin and chromogranin and negative for TTF1 and thyroglobulin. Congo red stain focally positive for amyloid	Focality: multifocal #1 Tumor site: right lobe Tumor Size: 3 cm Histologic type: MTC Margins: uninvolved Angioinvasion: no Lymphatic invasion: no ETE: no Regional LN: Involved: 0 Examined: 8 AJCC staging T2*N*0Mx Immunostaining: positive chromogranin, synaptophysin, and calcitonin #2 Tumor site: left lobe Tumor size: 0.8 cm Histological type: PTC classic subtype Margins: uninvolved Angioinvasion: no Lymphatic invasion: no ETE: no Regional LN: Involved: 1 Examined: 8 Largest metastatic deposit 0.3 cm ENE: no AJCC staging T1aN1aMx
Postoperative Laboratory testing (1-6 wk)	Calcitonin <5.0 pg/mL (normal range <5 pg/mL^b^) CEA 4.8 ng/mL (normal range ≤3.0 ng/mL)	Calcitonin <5.0 pg/mL (normal range <5 pg/mL^b^) CEA 0.8 ng/mL (normal range ≤3.0 ng/mL)	Calcitonin <5.0 pg/mL (normal range <5 pg/mL^b^) CEA 1.4 ng/mL (normal range ≤3.0 ng/mL)	Calcitonin = 23 pg/mL (∗∗normal range <5 pg/mL) CEA 198 ng/mL (normal range ≤3.0 ng/mL)
Last follow-up: laboratory testing and neck ultrasound	59 mo Calcitonin <5.0 pg/mL (normal range <5 pg/mL^b^) CEA 1.2 ng/mL (normal range ≤3.0 ng/mL) NED	72 mo Calcitonin <5.0 pg/mL (normal range <5 pg/mL^b^) CEA 0.5 ng/mL (normal range ≤3.0 ng/mL) NED	42 mo Calcitonin <5.0 pg/mL (∗∗normal range <5 pg/mL) CEA 1.0 ng/mL (normal range ≤3.0 ng/mL) NED	4 mo Calcitonin <5.0 pg/mL (∗∗normal range <5 pg/mL) CEA 4.9 ng/mL (normal range ≤3.0 ng/mL) NED

Abbreviations: AJCC = American Joint Committee on Cancer; CEA= carcinoembryonic antigen; ETE = extrathyroidal extension; ENE = extranodal extension; LN = lymph node; MTC = medullary thyroid cancer; NED = no evidence of disease; PTC = papillary thyroid carcinoma.

a Reference range for patients with intact thyroid.

b Reference range for patients post total thyroidectomy.

## Discussion

The diagnosis of MTC in clinical practice has been a challenge for several reasons. Due to its low incidence, many physicians are unfamiliar with the clinical presentation.^2^ The absence of specific ultrasound characteristics further contributes to the diagnostic challenge. In a retrospective cohort of 152 pathologically proven MTCs, the review of the preoperative ultrasound images, revealed that less than half were categorized as high suspicion nodules according to existing risk stratification systems, and less than two-thirds were recommended fine needle aspiration (FNA).^4^ In our series, 2 cases presented as solid solitary hypoechoic nodules, one was solid isoechoic and the fourth was a complex, cystic nodule with a solid hypoechoic component.

The limited sensitivity of FNAC for the diagnosis of MTC has been well acknowledged. In the largest meta-analysis to date, which included 641 MTC lesions that had undergone FNAC, the pooled detection rate of MTC was 56.4% (12.5% to 88.2%). In most cases, MTC was misclassified as oncocytic or follicular neoplasms, such as in 3 of our cases.^8^ The cytologic appearance of MTC is variable.^9^ (Fig. 1) Typical features include dispersed or loosely cohesive population of cells with various degrees of cellularity. The cells are typically uniform in size, and can have epithelioid, plasmacytoid, polygonal, or spindled appearance. Nuclei are round to oval, eccentric with coarse granular chromatin and nuclear grooves are typically absent. Cytoplasm is often granular and amphophilic, with azurophilic granules present in up to 50% of cases.^6^^,^^7^ Background amyloid can be observed in one-third of MTC cases;^,^ however, its close resemblance to colloid especially on Thin Prep liquid based cytology slide preparation may result in missing this subtle clue to diagnosis of MTC.^6^ Furthermore, intranuclear pseudoinclusions are sometimes observed in MTC which is often considered diagnostic for papillary thyroid carcinoma.^7^ Both MTC and oncocytic neoplasms can present as hypercellular discohesive cells with eccentric nuclei and granular cytoplasm. Although oncocytes typically have a prominent macronucleolus instead of the salt-and pepper chromatin seen in most MTCs, this feature is not always evident in cytology samples.^6^^,^^7^

**Fig:**
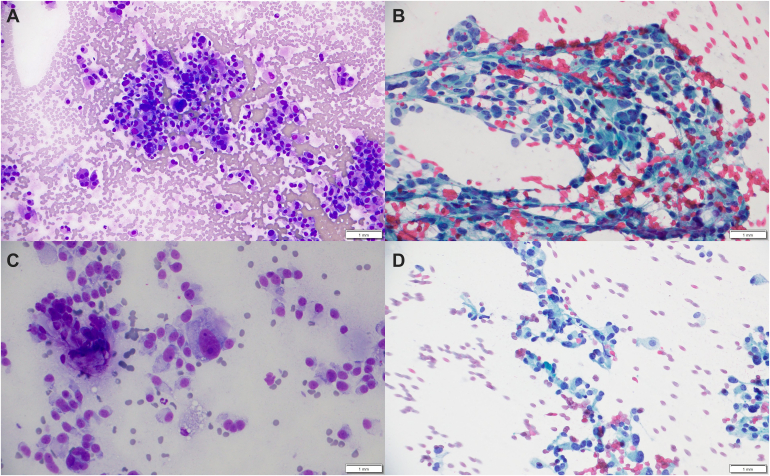
*A*. Modified Wright-Giemsa stain, intermediate magnification: Clusters of Hurthle cells with pleomorphic nuclei are present. Loose, single cells are present at the periphery. *B*, Papanicolaou stain, intermediate magnification: Loosely clustered epithelioid cells with abundant cytoplasm. Rare spindle cells are present. The nuclei contain inconspicuous nucleoli. *C*, Modified Wright-Giemsa stain, high magnification: Pleomorphic nuclei with nucleoli present with ample cytoplasm and maintained nuclear: cytoplasmic ratio. *D*, Papanicolaou stain, intermediate magnification: Loose clusters of epithelioid cells present with minimal cytologic atypia and ample cytoplasm.

Several ancillary diagnostic modalities have been proposed to mitigate these cytologic challenges.^10^ For cytology cases deemed inconclusive or potentially indicative of MTC, the ATA recommends measurement of calcitonin in the washout fluid, and immunostaining for relevant biomarkers (chromogranin, calcitonin, carcinoembryonic antigen) along with confirming lack of thyroglobulin staining.^1^ Likewise, European Thyroid Association considers that calcitonin washout assessment can be useful in some small nodules with normal serum calcitonin but cytological suspicion of MTC, or in cases of elevated serum calcitonin.^11^ In a study of 87 thyroid nodules, calcitonin assay in FNA washout outperformed FNAC in terms of diagnostic accuracy (98.85% vs 61.90%), sensitivity (98.55% vs 55.07%), and specificity (100% vs 97.44%).^12^ A systematic review comparing FNAC with calcitonin measurement in the FNA washout fluid found that FNA washout was significantly more sensitive than cytology in detecting MTC and recommended its standardized use when cytology is suspicious for MTC while acknowledging that lack of specific cutoff points and possible false positive results in the setting of C-cell hyperplasia represent the main current limitations.^13^ Immunocytochemistry of FNAC samples was recommended to disambiguate nodules with indeterminate cytologic characteristics, while a routine second opinion from another pathologist is a common strategy in many large institutions but may not be feasible in smaller practices. In a study of 331 FNAC cases, a second opinion reduced the need for diagnostic thyroidectomy in 25% of cases without increase in false negatives.^14^

Current ATA and National Comprehensive Cancer Network guidelines recommend use of molecular testing for further characterization of thyroid nodules classified as Bethesda category IV (follicular neoplasms), and Bethesda category III (AUS).^15^^,^^16^ However, for Bethesda category III nodules, current recommendations also include the option of repeating FNAC, diagnostic lobectomy or surveillance, resulting in significant differences in clinical practices among thyroidologists.^15^^,^^17^

The most commonly used molecular testing platforms for diagnosing indeterminate thyroid nodules commercially available in the United States include Afirma GSC, Thyroseq v3, and ThyGeNext/ThyraMIR.^18^^,^^19^ Afirma GSC is a genomic sequencing classifier that uses RNA whole transcriptome sequencing and machine learning to predict the malignant potential of a thyroid nodule.^18^^,^^19^ Thyroseq v3 is a multigene genomic classifier that utilizes next gen sequencing analysis conducted for 4 classes of genetic alterations in 112 genes.^18^^,^^19^ ThyGeNext/ThyraMIR is a two-step platform which includes a panel testing for 10 gene mutations and 38 gene fusions present in common malignancies, followed by a gene expression analysis of 10 miRNA genes, to predict how weak driver mutations impact nodule behavior.^18^^,^^19^

While the literature specifically evaluating the performance of molecular testing platforms in the diagnosis of MTC is limited, all platforms include genetic markers relevant for MTC and have demonstrated high diagnostic accuracy in cytology samples.^20^, ^21^, ^22^

An analytical and clinical validation study of the ThyraMIR platform for diagnosis of MTC in cytology samples demonstrated 100% accuracy (95% CI 99% to 100%). Likewise, Afirma MTC RNA-sequencing classifier showed 100% sensitivity (21/21 MTC FNAC samples correctly classified as positive) and 100% specificity (190/190 non-MTC FNAC samples correctly classified as negative), with histopathological confirmation.^21^ In a validation study for ThyroSeq v3, of 238 surgically removed tissues, 15/15 (100%) were correctly classified as MTCs.^22^ ThyroSeq v3 correctly diagnosed all 4 cases in our case series as MTC.^21^ In addition to the benefit represented by the correct classification, recent advancements in understanding of genetic alterations present in thyroid cancers have therapeutic implications, arguing further in favor of the use of molecular testing.^18^ A recent analysis of 50 734 thyroid nodules classified as Bethesda categories III-VI that were evaluated using ThyroSeq v3, included 124 (0.2%) cases of MTC, and found that all these nodules demonstrated high expression of calcitonin and other neuroendocrine genes consistent with MTC. In addition, the most common mutations reported were in the *RET* gene (67 nodules) followed by *HRAS*, *KRAS* and *BRAF* in 26, 6, and 3 nodules respectively, while 16 nodules displayed copy number alterations with other mutations.^23^

It is important to recognize that in absence of a diagnosis of MTC, asymptomatic patients in these categories may elect surveillance with the added risk of being lost to follow up, with negative long-term consequences. In our series, all 4 cases were correctly diagnosed with molecular testing and confirmed preoperatively by elevated serum calcitonin. In absence of the correct diagnosis, 3 of the patients would have been recommended lobectomy and one may have undergone surveillance, as per the thyroid nodule/differentiated thyroid cancer guidelines. Instead, all 4 patients were treated according to the standard of care for MTC and had no biochemical or anatomical evidence of disease at the last follow up.

## Conclusion

As MTC has been long recognized as a cytologic diagnostic conundrum, the use of ancillary testing, specifically molecular testing platforms, could have a direct impact on patient prognosis as an early diagnosis would significantly increase the likelihood of complete tumor resection and cure.

## Data Availability Statement

Data sharing is not applicable to this article as no additional datasets were generated or analyzed during the current study.

## Patient Consent

Patient information has been collected under IRB 23-002031.

## Disclosure

The authors have no conflicts of interest to disclose.

## References

[1] Wells S.A., Asa S.L., Dralle H. (2015). Revised American Thyroid Association guidelines for the management of medullary thyroid carcinoma. Thyroid.

[2] Thomas C.M., Asa S.L., Ezzat S., Sawka A.M., Goldstein D. (2019). Diagnosis and pathologic characteristics of medullary thyroid carcinoma-review of current guidelines. Curr Oncol.

[3] Shao Y., Li G., Wei T. (2022). Distant metastasis in medullary thyroid carcinoma: clinical outcomes and implications of T stage. Clin Endocrinol (Oxf).

[4] Matrone A., Gambale C., Biagini M., Prete A., Vitti P., Elisei R. (2021). Ultrasound features and risk stratification systems to identify medullary thyroid carcinoma. Eur J Endocrinol.

[5] Trimboli P., Nasrollah N., Amendola S. (2012). Should we use ultrasound features associated with papillary thyroid cancer in diagnosing medullary thyroid cancer?. Endocr J.

[6] Pusztaszeri M.P., Bongiovanni M., Faquin W.C. (2014). Update on the cytologic and molecular features of medullary thyroid carcinoma. Adv Anat Pathol.

[7] Rossi E.D., Adeniran A.J., Faquin W.C. (2019). Pitfalls in thyroid cytopathology. Surg Pathol Clin.

[8] Trimboli P., Treglia G., Guidobaldi L. (2015). Detection rate of FNA cytology in medullary thyroid carcinoma: a meta-analysis. Clin Endocrinol (Oxf).

[9] Pusztaszeri M.P., Maleki Z. (2025). The diagnostic challenges of medullary thyroid carcinoma: a practical guide for cytopathologists. Cancer Cytopathol.

[10] Workman A.D., Soylu S., Kamani D. (2021). Limitations of preoperative cytology for medullary thyroid cancer: proposal for improved preoperative diagnosis for optimal initial medullary thyroid carcinoma specific surgery. Head Neck.

[11] Durante C., Hegedus L., Czarniecka A. (2023). 2023 European Thyroid Association Clinical Practice Guidelines for thyroid nodule management. Eur Thyroid J.

[12] Liu Z., Zhou W., Han R. (2021). Cytology versus calcitonin assay in fine-needle aspiration biopsy wash-out fluid (FNAB-CT) in diagnosis of medullary thyroid microcarcinoma. Endocrine.

[13] Trimboli P., Giannelli J., Marques B., Piccardo A., Crescenzi A., Deandrea M. (2022). Head-to-head comparison of FNA cytology vs. calcitonin measurement in FNA washout fluids (FNA-CT) to diagnose medullary thyroid carcinoma. A systematic review and meta-analysis. Endocrine.

[14] Davidov T., Trooskin S.Z., Shanker B.A. (2010). Routine second-opinion cytopathology review of thyroid fine needle aspiration biopsies reduces diagnostic thyroidectomy. Surgery.

[15] Haugen B.R., Alexander E.K., Bible K.C. (2016). 2015 American Thyroid Association Management Guidelines for adult patients with thyroid nodules and differentiated thyroid cancer: the American thyroid Association Guidelines task force on thyroid nodules and differentiated thyroid cancer. Thyroid.

[16] Haddad R.I., Bischoff L., Applewhite M. (2025). NCCN Guidelines(R) insights: Thyroid Carcinoma, Version 1.2025. J Natl Compr Canc Netw.

[17] Ali S.Z., Baloch Z.W., Cochand-Priollet B., Schmitt F.C., Vielh P., VanderLaan P.A. (2023). The 2023 bethesda System for reporting thyroid cytopathology. Thyroid.

[18] Hannoush Z.C., Ruiz-Cordero R., Jara M., Kargi A.Y. (2024). Current State of molecular cytology in thyroid nodules: platforms and their diagnostic and Theranostic utility. J Clin Med.

[19] Dettmer M.S. (2025). The actual and future role of molecular tests in thyroid pathology. Virchows Arch.

[20] Ciarletto A.M., Narick C., Malchoff C.D. (2021). Analytical and clinical validation of pairwise microRNA expression analysis to identify medullary thyroid cancer in thyroid fine-needle aspiration samples. Cancer Cytopathol.

[21] Randolph G.W., Sosa J.A., Hao Y. (2022). Preoperative identification of Medullary Thyroid Carcinoma (MTC): clinical validation of the Afirma MTC RNA-Sequencing classifier. Thyroid.

[22] Nikiforova M.N., Mercurio S., Wald A.I. (2018). Analytical performance of the ThyroSeq v3 genomic classifier for cancer diagnosis in thyroid nodules. Cancer.

[23] Chiosea S., Hodak S.P., Yip L. (2023). Molecular profiling of 50 734 bethesda III-VI thyroid nodules by ThyroSeq v3: implications for personalized management. J Clin Endocrinol Metab.

